# Dynamics of Vascular Protective and Immune Supportive Sphingosine-1-Phosphate During Cardiac Surgery

**DOI:** 10.3389/fimmu.2021.761475

**Published:** 2021-10-20

**Authors:** Gillis Greiwe, Eileen Moritz, Katharina Amschler, Annika Poppe, Harun Sarwari, Axel Nierhaus, Stefan Kluge, Hermann Reichenspurner, Christian Zoellner, Edzard Schwedhelm, Günter Daum, Björn Tampe, Martin Sebastian Winkler

**Affiliations:** ^1^ Department of Anesthesiology, Center of Anesthesiology and Intensive Care Medicine, University Medical Center Hamburg-Eppendorf, Hamburg, Germany; ^2^ Institute of Pharmacology, Department of General Pharmacology, University Medicine Greifswald, Greifswald, Germany; ^3^ German Center for Cardiovascular Research (DZHK), Partner Site Greifswald, Greifswald, Germany; ^4^ Institute of Clinical Pharmacology and Toxicology, University Medical Center Hamburg-Eppendorf, Hamburg, Germany; ^5^ Department of Dermatology, Venereology and Allergology, University Medical Center, Göttingen, Germany; ^6^ Clinic and Policlinic for Anesthesiology and Intensive Care Medicine, University Medicine Rostock, Rostock, Germany; ^7^ Department of Cardiovascular Surgery, University Heart Center Hamburg, Hamburg, Germany; ^8^ Department of Intensive Care Medicine, Center of Anesthesiology and Intensive Care Medicine, University Medical Center Hamburg-Eppendorf, Hamburg, Germany; ^9^ German Center for Cardiovascular Research (DZHK), Partner Site Hamburg/Kiel/Lübeck, Hamburg, Germany; ^10^ Department of Vascular Medicine, University Heart and Vascular Center Hamburg-Eppendorf, Hamburg, Germany; ^11^ Department of Nephrology and Rheumatology, University Medical Center Göttingen, Göttingen, Germany; ^12^ Department of Anesthesiology and Intensive Care, University Medical Center Göttingen, Göttingen, Germany

**Keywords:** sphingosine-1-phosphate, systemic inflammation, sepsis, cardiac surgery, heparin, SIRS

## Abstract

**Introduction:**

Sphingosine-1-phosphate (S1P) is a signaling lipid and crucial in vascular protection and immune response. S1P mediated processes involve regulation of the endothelial barrier, blood pressure and S1P is the only known inducer of lymphocyte migration. Low levels of circulatory S1P correlate with severe systemic inflammatory syndromes such as sepsis and shock states, which are associated with endothelial barrier breakdown and immunosuppression. We investigated whether S1P levels are affected by sterile inflammation induced by cardiac surgery.

**Materials and Methods:**

In this prospective observational study we included 46 cardiac surgery patients, with cardiopulmonary bypass (CPB, n=31) and without CPB (off-pump, n=15). Serum-S1P, S1P-sources and carriers, von-Willebrand factor (vWF), C-reactive protein (CRP), procalcitonin (PCT) and interleukin-6 (IL-6) were measured at baseline, post-surgery and at day 1 (POD 1) and day 4 (POD 4) after surgical stimulus.

**Results:**

Median S1P levels at baseline were 0.77 nmol/mL (IQR 0.61-0.99) and dropped significantly post-surgery. S1P was lowest post-surgery with median levels of 0.37 nmol/mL (IQR 0.31-0.47) after CPB and 0.46 nmol/mL (IQR 0.36-0.51) after off-pump procedures (P<0.001). The decrease of S1P was independent of surgical technique and observed in all individuals. In patients, in which S1P levels did not recover to preoperative baseline ICU stay was longer and postoperative inflammation was more severe. S1P levels are associated with its sources and carriers and vWF, as a more specific endothelial injury marker, in different phases of the postoperative course. Determination of S1P levels during surgery suggested that also the anticoagulative effect of heparin might influence systemic S1P.

**Discussion:**

In summary, serum-S1P levels are disrupted by major cardiac surgery. Low S1P levels post-surgery may play a role as a new marker for severity of cardiac surgery induced inflammation. Due to well-known protective effects of S1P, low S1P levels may further contribute to the observed prolonged ICU stay and worse clinical status. Moreover, we cannot exclude a potential inhibitory effect on circulating S1P levels by heparin anticoagulation during surgery, which would be a new pro-inflammatory pleiotropic effect of high dose heparin in patients undergoing cardiac surgery.

## Introduction

Cardiac surgery is still associated with a high early perioperative in-hospital mortality (1, 2). Surgery related mortality has partially been attributed to the surgery induced acute inflammatory response. In this context, cardiopulmonary bypass (CPB) induces severe inflammation and is associated with higher risk of organ failure such as acute kidney injury (AKI), which increases the risk of not surviving the hospital stay by 2-fold (3–5). Although techniques such as off-pump procedures may reduce postoperative mortality, the acute inflammatory response after cardiac surgery remains challenging due to the massive cardiovascular stress after surgery (6). Mechanisms of surgery induced inflammation and other causes of systemic inflammation are very similar. For example, the clinical presentation of sepsis patients compared to patients with severe postoperative inflammation after cardiac surgery is indistinguishable and hemodynamic instability, volume deficiency and lactate acidosis are leading symptoms (7, 8). Hallmarks are hypotension caused by vasoplegia, edema formation caused by endothelial barrier disruption and uncontrolled cytokine secretion with an increased vulnerability for secondary infections. All of these processes are potentially regulated by the vascular protective G-protein coupled signalling lipid sphingosine-1-phosphate (S1P) (9). S1P supports crucial functions relevant in inflammation and in the control of hyperinflammatory states: I) Regulation of the endothelial cell (EC) barrier: S1P is fundamental for EC barrier stabilization by regulating the EC cytoskeleton (10, 11). *In-vivo* models have demonstrated a robust stabilizing effects of S1P preventing lung edema and microvascular permeability induced by toxins (endotoxin, lipopolysaccharide) or high-volume ventilation (12, 13). II) Recruitment of lymphocytes and host response: S1P is the only known inducer of lymphocyte migration from lymphatic organs into peripheral blood. Mice with depleted S1P levels are lymphopenic (14–16). This effect of S1P on the immunity is induced by FTY-720 (Fingolimod), which is a standard of care in Multiple sclerosis. Moreover, S1P agonists are effectful inhibitors of viral induced inflammation in mice infected with influenza (17). Finally, S1P signaling may depend on its main carrier, high-density lipoprotein (HDL) or albumin (18).

There is growing evidence that S1P levels are low in patients with sepsis induced systemic inflammation and that low S1P levels correlate with a higher mortality and morbidity (19–22). Moreover, decreased S1P levels are predictive of hyperinflammatory shock with similar precision as the sequential organ failure assessment (SOFA) (20). Manipulating S1P-controlled processes by S1P substitution or S1P-receptor activation are discussed as therapeutic options to attenuate the systemic inflammatory response. These effects are within the range of expectancy due to the well-known activities of S1P on endothelial integrity, the immune response and as a pro-survival factor (20, 23–25). Considering the observations in human systemic inflammation and current experimental data, we aimed to investigate S1P in patients undergoing cardiac surgery induced inflammation.

## Materials and Methods

### Study Design and Subjects

Forty-six adult patients (age >18 years) scheduled for elective major cardiac surgery, with or without CPB, at the University Heart and Vascular Center at the University Medical Center Hamburg-Eppendorf, Germany, were enrolled in the study after written informed consent was obtained. Non-inclusion criteria was a preexisting infection or the suspicion thereof based on laboratory results. Treatment of patients was entirely left to the discretion of the caring surgeon, anesthesiologist and intensive care provider. After insertion of an arterial radial catheter by the caring anesthesiologist prior to anesthesia induction, the first blood samples (7,5 ml volume) were drawn to define a preoperative baseline. Blood samples were repeatedly drawn after surgery, before transfer of the patient to the intensive care unit (ICU), on the first postoperative day (POD1) and on the fourth postoperative day (POD4). In six patients, additional serum blood samples were drawn at skin incision, before CPB and every 30 minutes during CPB. Laboratory tests included S1P concentrations in blood, potential sources of S1P (red blood cells (RBC), platelets), coagulation tests (partial thromboplastin time (PTT), international normalized ratio (INR), inflammatory markers (interleukin-6 (IL-6), procalcitonin (PCT), c-reactive protein (CRP)), von-Willebrand factor antigen (vWF : AG), fibrinogen), potential S1P carriers (high-density lipoprotein (HDL), low-density lipoprotein (LDL), albumin), bilirubin and creatinine. Except for S1P, all other laboratory measurements were performed with routine patients’ diagnostic tests with appropriate quality controls and clinical standards (Institute of Clinical Chemistry and Laboratory Medicine at the University Medical Center Hamburg-Eppendorf). In addition to basic patient characteristics and performed surgical procedure we documented the following clinical parameters during the perioperative phase: duration of ECC and aortal cross-clamping time, overall time of surgery, fluid balance, dosage of vasoactive drugs, perioperative administration of blood products, length of ICU stay and the Sequential Organ Failure Assessment (SOFA) score on POD1.

### Serum Preparation and S1P Measurements

Blood serum was obtained by coagulation for at least 60 min, cleared by centrifugation and immediately frozen and stored at −80°C until further use. According to the manufacturer’s protocol we used serum tubes filled with kaolin or silicate coated beads to guarantee a full coagulation within the expected time of at least 60 min (S-monovette™, Sarstedt, Nümbrecht, Germany). S1P measurements were performed by liquid chromatography-tandem mass spectrometry (LC-MS/MS) as previously described (26). Briefly, after the addition of internal standard (1 nmol/mL S1P-d7, Avanti Polar Lipids, Alabaster, AL, USA), 20 µL-aliquots of serum were de-proteinated by the addition of 180 µL acetonitrile/water (80/20, vol/vol). Extracts were cleared by centrifugation and subjected to reverse-phase chromatography on a Zorbax SB-C8 column (2.1 × 50 mm; Agilent Technologies, Santa Clara, CA, USA) at a flow rate of 0.35 mL/min. S1P was eluted by a binary gradient over six minutes (methanol/acetonitrile/0.1% formic acid, 2.5/2.5/95, vol/vol/vol to methanol/acetonitrile/0.1% formic acid, 30/30/40, vol/vol/vol) and quantified by LC-MS/MS (Varian L1200 MS/MS, Agilent Technologies, Waldbronn, Germany) in the multiple reaction mode. Four level calibration curves and two levels of quality controls (QCs) were included. Imprecision of the method was determined to 8% and 9% for QC-low and QC-high samples, respectively (26).

### Statistical Analysis

The primary variable was S1P concentration in nanomoles (nmol) per milliliter (mL). Differences between groups were tested for significance by using non-parametric tests: Mann–Whitney U test for two groups and Kruskal-Wallis analysis of variance (ANOVA) for more than two groups. Data are presented as median with interquartile range and *in-vitro* experiments as mean with standard error of the mean. Correlations and multivariate regression analysis were performed either by the Spearmans’ rank correlation test and multivariate regression analysis using SPSS (version 21; IBM Corporation, Armonk, NY, USA). In addition, a Kaplan-Meier curve was generated for subgroup analysis. For all tests, a P-value of less than 0.05 was considered significant. Statistical analyses were performed by using SPSS or GraphPad Prism (version 8.4.2, GraphPad, La Jolla, CA, USA) with guidance from members of the Department of Medical Biometry and Epidemiology at the University Hospital Hamburg-Eppendorf.

## Results

### Circulatory S1P Levels Decrease, Whereas Inflammatory Markers Increase After Surgery

**Figure 1:**
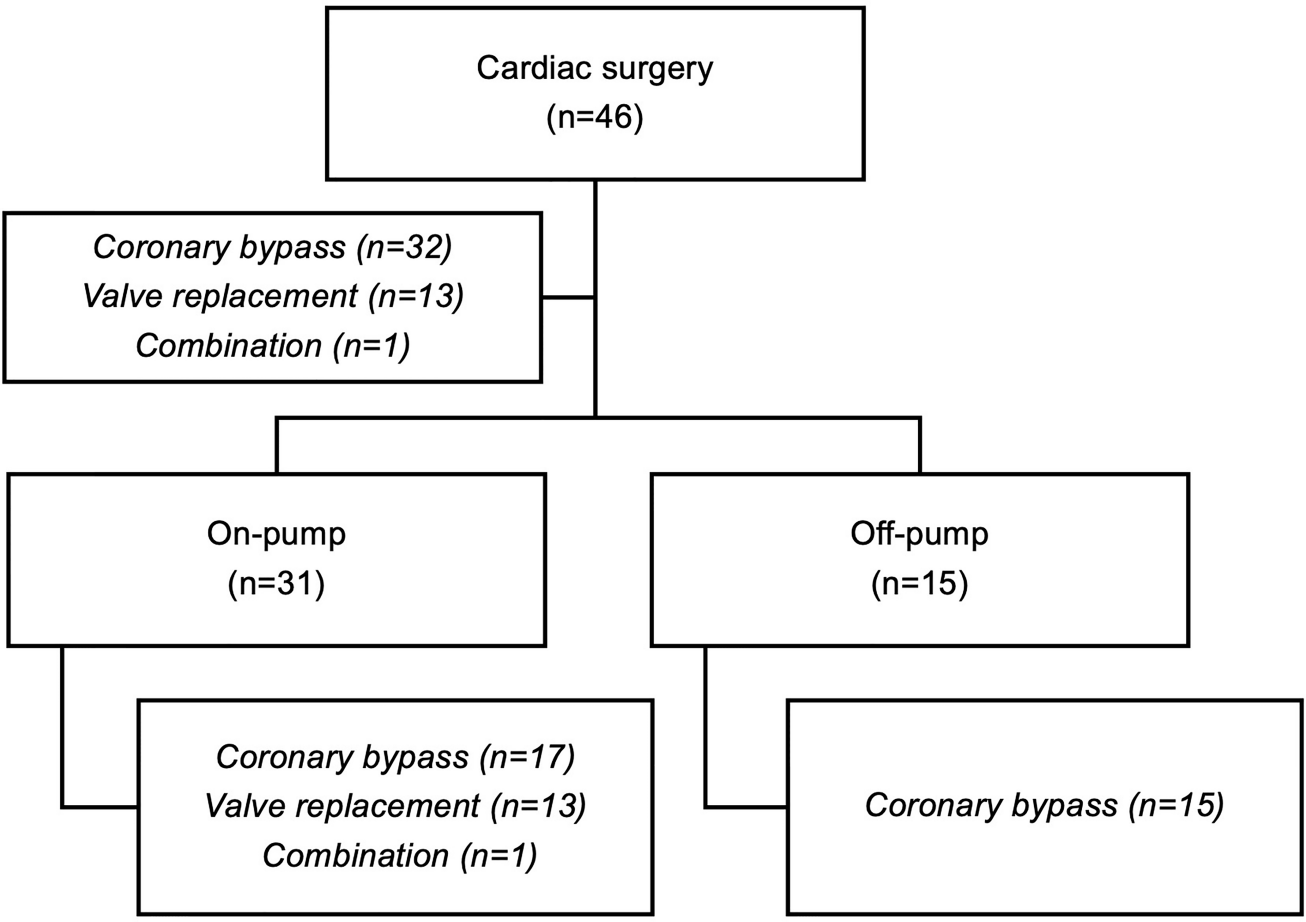
Flow-chart of the included study participants.

**Figure 2:**
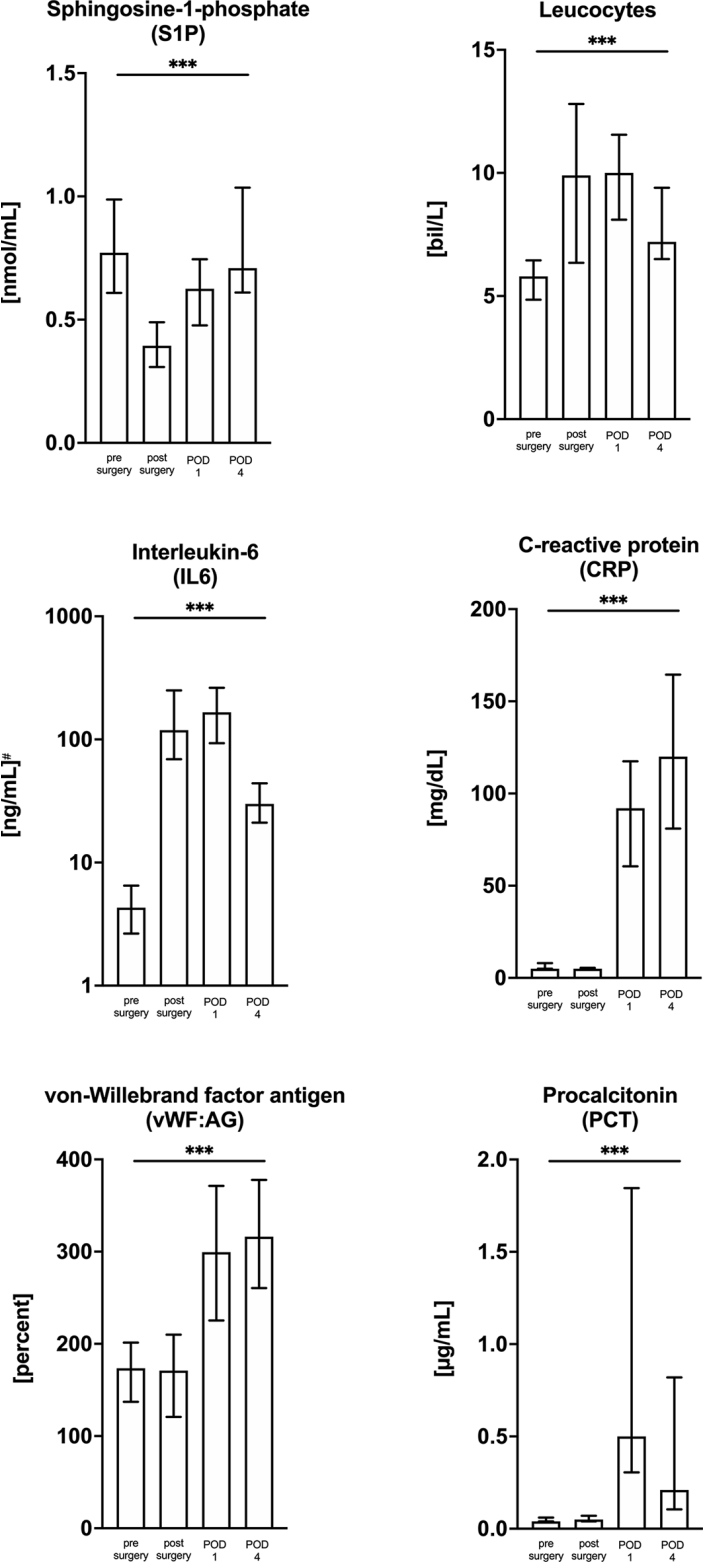
Kinetic of S1P levels and other inflammatory markers in cardiac surgery patients from pre-surgery levels (baseline) up to day four after surgery. All markers were measured in 46 patients admitted to hospital for cardiac surgery. Among all measured parameters only S1P decreased after surgical intervention. The unity and scale have been adjusted for each marker and data are presented as median with interquartile range (IQR). The statistic is showing the ANOVA Kruskal-Wallis test for trend analysis. ***p < 0.001. POD, postoperative day.

We included 46 patients admitted for elective cardiac surgery. Thirty-one (67.4%) patients underwent on-pump procedures for coronary artery bypass surgery (n=17), valve replacement/reconstruction (n=13) or a combination of coronary artery bypass surgery and valve replacement (n=1). Fifteen patients (32.6%) underwent off-pump procedures (off-pump coronary artery bypass surgery). All patients were admitted to hospital because of coronary heart disease (CHD) and received the preoperative standard of care for CHD. The chart in **Figure 1** summarizes the included patient groups. We compared S1P levels and five inflammatory markers to investigate whether levels were altered in relation to pre-surgery levels and **Table 1** is showing basic patient characteristics. S1P was the only laboratory characteristic, which significantly decreased compared to pre-surgery baseline levels (**Figure 2**). The lowest S1P concentrations were found post-surgery when patients were transferred to the intensive care unit (ICU). All other markers showed a contrary trend with significant peak levels either directly post-surgery (leucocytes), on postoperative day (POD) 1 (procalcitonin/PCT, interleukin-6/IL6), or POD4 (von-Willebrand-factor:AG/vWF : AG, C-reactive protein/CRP, **Figure 2**).

**Table 1:** Baseline patient’s characteristics.

Parameter	All patients	On-pump	Off-pump	P-value
Number of patients, n	46	31	15	N/A
Age, y	70 (62-75)	68 (57-74)	72 (67-75)	ns
Male/Female, n/n	34/12	21/10	13/2	<0.05
S1P, nmol/mL	0.77 (0.61-0.99)	0.85 (0.66-1.03)	0.67 (0.54-0.76)	<0.05
S1P source and vWF : AG				
Erythrocytes, 10^6^/µL	4.25 (3.79-4.53)	4.27 (3.99-4.52)	4.07 (3.48-4.67)	ns
Platelets, 10^3^/µL	234 (195-263)	237 (198-300)	200 (181-248)	ns
vWF : AG, %	174 (137-201)	162 (126-195)	193 (155-253)	<0.05
S1P carriers				
HDL, mg/dL	43.0 (36.-57.0)	44.5 (33.5-59.0)	43.0 (38.5-51.5)	ns
Albumin, g/L	34.0 (31.0-36.0)	34.0 (32.0-36.0)	33.0 (30.0-35.5)	ns
Inflammatory marker				
Leucocytes, 10^3^/µL	5.8 (4.9-6.5)	5.9 (4.9-6.6)	5.6 (4.8-6.3)	ns
IL-6, ng/L	4.3 (2.7-6.5)	4.4 (2.2-6.1)	4.3 (3.1-9.6)	ns
CRP, mg/L^#^	5 (5-8)	5 (5-7)	5 (5-10)	ns
PCT, ng/mL	0.04 (0.04-0.06)	0.04 (0.04-0.06)	0.04 (0.035-0.055)	ns

Data are presented as median and interquartile range (IQR); CRP, C-reactive protein; vWF, AG, von Willebrand-Factor antigen; IL-6, Interleukin-6; PCT, Procalcitonin; N/A, not applicable; ns, not significant; ^#^Detection limit 5 mg/dL.

### Cardiac Surgery Disrupts Serum-S1P Levels Irrespective of the Use of CPB

**Figure 3:**
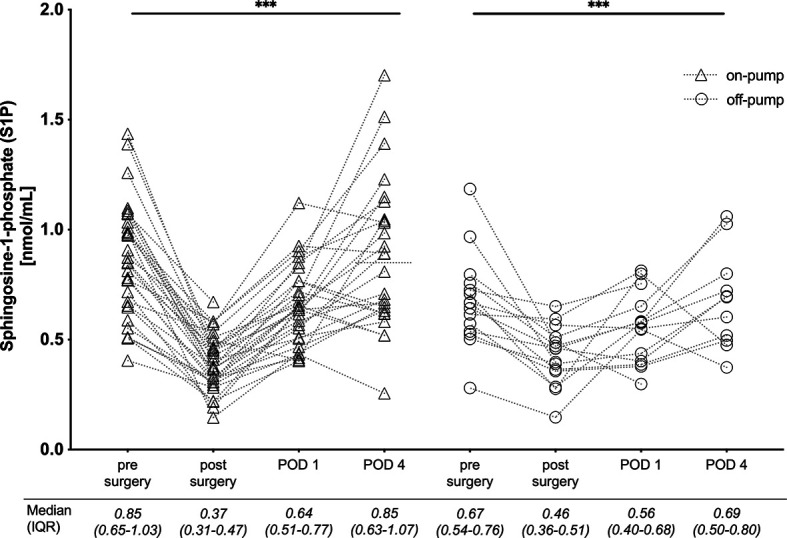
Comparison of S1P levels in 46 cardiac surgery patients operated with cardiopulmonary bypass (on-pump) and without (off-pump). Individual serum-S1P levels are plotted at four time points: pre-surgery, post-surgery with admission to intensive care unit, on day one (POD 1) and four (POD 4) after surgery. The median and interquartile range (25^th^ to 75^th^ percentile) of measured serum-S1P are listed below. The trend for serum S1P was significant for both groups and lowest S1P levels were reached post-surgery. Median post-surgery levels were not different when comparing on- versus off-pump patients. The statistic within the graph is showing ANOVA Kruskal-Wallis test for trend analysis for the complete observational period. ***p < 0.001. POD, postoperative day; IQR, interquartile range.

In order to investigate the influence of CPB on S1P kinetics we defined two groups (**Figure 1**): patients operated with support of CPB (referred to as on-pump), and patients operated without CPB (referred to as off-pump group). Basic patient characteristics and baseline inflammatory markers were compared between the two groups (**Table 1**). We found the same S1P kinetics in both groups with significant lowest levels observed post-surgery (**Figure 3**). S1P levels dropped by 58% in the on-pump and 31% in the off-pump group (**Figure 3**). Regardless of baseline levels being high or low, patients reached their individual nadir post-surgery with lowest S1P levels of 0.37 nmol/mL in the on-pump group and 0.46 nmol/mL in the off-pump group (**Figure 3**). However, the difference between these two levels was not significant. Taken together, the lowest S1P levels post-surgery were independent of the use of CPB.

### Changes of Serum-S1P Levels Are Associated With S1P Sources and Carriers

**Figure 4:**
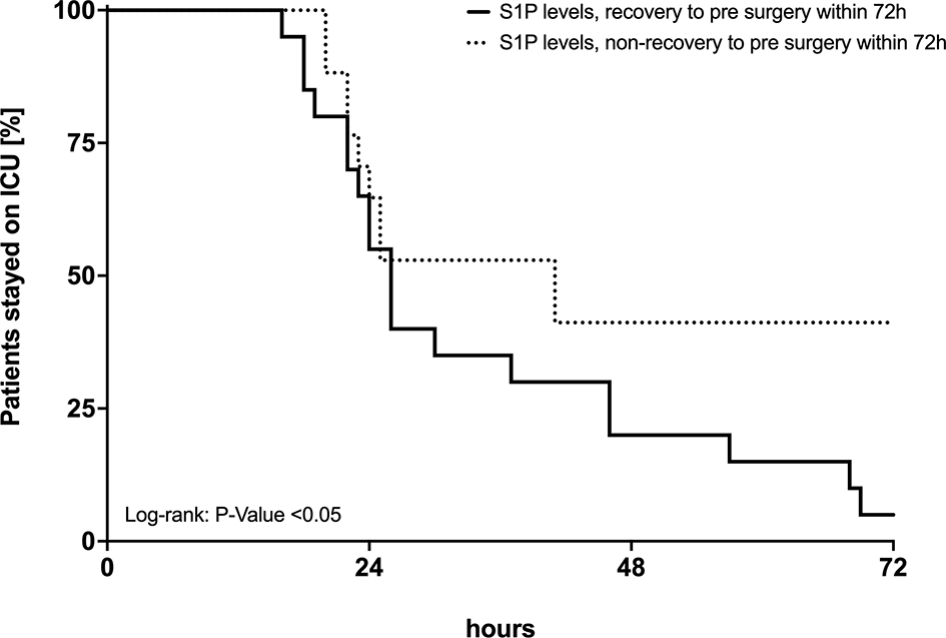
Length of ICU stay in two patient groups. Patients in which sphingosine-1-phosphate (S1P) levels recovered and patients in which S1P levels did not recover to pre-surgery levels. The x-axis shows ICU stay in hours, the y-axis the percentage of patients remaining on ICU for further observation and treatment. Differences between the two groups were tested by Log-rank-test for comparison of survival curves.

To identify potential parameters that define the U-shaped course of S1P levels during treatment, a multivariate regression analysis was performed for three perioperative phases: intraoperative, early recovery (POD 1) and late recovery (POD 4). We included S1P sources, red blood cells (RBC) and platelets, S1P carriers, serum albumin, high-density lipoprotein (HDL) and a marker for endothelial cell injury, vWF : AG. Intraoperative loss of S1P was associated with RBC and platelets depletion, whereas the increase of S1P levels on POD 1 and POD 4 was dependent on albumin, HDL and vWF : AG activity (**Table 2**). Next, we investigated whether postsurgical S1P levels were predictive for patient outcome. Patients were divided into two groups: S1P levels increasing to reach individual pre-surgery levels, and S1P levels remaining low. Both groups were compared using the sequential organ failure assessment (SOFA) score on POD1, fluid balance within the first 24 hours after surgery and length of ICU stay. Patients with a full recovery of S1P levels presented with a lower SOFA score (*p<0.05*), had a reduced volume uptake (not significant) and stayed significantly shorter on ICU (*p<0.05*) (**Figure 4** and **Table 3**).

**Table 2:** Multivariate regression analysis with sphingosine-1-phosphate (S1P) as dependent variable for different perioperative phases.

Parameter	Intraoperative	P-value	POD 1	P-value	POD 4	P-value
	Regression-coefficient (CI 95%)*		Regression-coefficient (CI 95%)*		Regression-coefficient (CI 95%)*	
Erythrocytes, 10^6^/µL	+0.307 (0.009 - 0.284)	<0.05	+0.073 (-0.149 - 0.212)	ns	-0.013 (-0.165 - 0.152)	ns
Platelets, 10^3^/µL	+0.404 (0.001 - 0.003)	<0.01	+0.121 (0.001 - 0.002)	ns	+0.271 (-0.001 - 0.002)	ns
vWF : AG, %	-0.134 (0.001 - 0.001)	ns	+0.195 (-0.001 - 0.001)	ns	-0.523 (0.003 - 0.001)	<0.01
HDL, mg/dL	+0.017 (-0.009 - 0.009)	ns	-0.068 (-0.011 - 0.008)	ns	-0.404 (-0.023 - 0.001)	<0.05
Albumin, g/L	+0.106 (-0.016 - 0.026)	ns	+0.345 (0.001 - 0.027)	<0.05	+0.521 (0.006 - 0.064)	<0.05
*Model-R^2#^*	*0.46*	*N/A*	*0.24*	*N/A*	*0.50*	*N/A*

Multivariate linear regression with S1P as dependent variable and S1P sources and carriers has been performed to predict S1P during the intraoperative phase, on day one (POD 1) and four (POD 4) after surgery. *Standardized regression coefficient (standard beta) is shown together with confidence interval (95% CI). ^#^R-square of the model is shown to demonstrate goodness-of-fit. vWF, AG, von Willebrand-Factor antigen; HDL, high-density lipoprotein; N/A, not applicable; ns, not significant.

**Table 3:** Comparison of outcome parameters.

Parameter	Full recovery of S1P	No recovery of S1P	P-value
Number of patients, n	23	20	N/A
SOFA	3 (2-3)	4 (3-4)	<0.05
ICU stay, h	24 (19-41)	41 (23-69)	<0.05
Balance, 24h post-surgery	700 (50-1800)	850 (550-1775)	ns

The study group was divided into two subgroups depending on whether serum S1P levels after surgery reached pre-surgery levels (full recovery of S1P) or not (no recovery of S1P). Data are presented as median, interquartile range (IQR); Groups were compared using non-parametric Mann-Whitney-U test. N/A, not applicable; ns, not significant.

### Serum-S1P Levels Drop Before the Start of CPB

**Figure 5:**
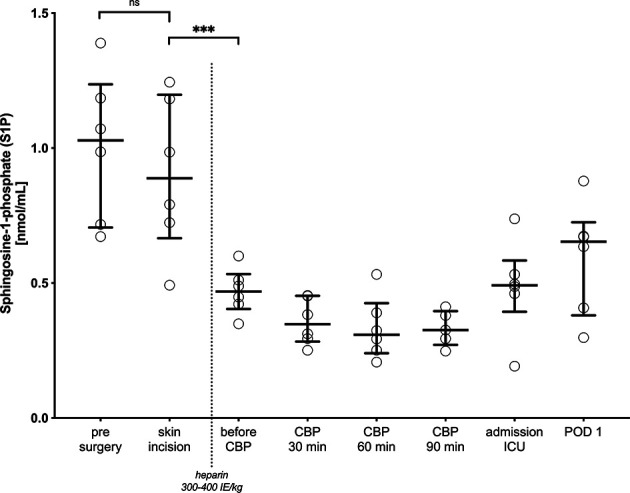
Intraoperative kinetic of S1P levels. Blood was drawn and analyzed every 30 minutes when cardiopulmonary bypass (CPB) had started, post-surgery, when patients were admitted to the intensive care unit (ICU), and 24h after surgery. Dots are individual serum-S1P concentrations and bars represent median levels together with interquartile range (IQR). A non-parametric paired t-test was performed to compare serum-S1P levels pre-surgery with levels before CPB at skin incision, before and after administration of intravenous unfractionated heparin (300-400 IE/kg) to reach the necessary anticoagulatory effect for CPB. While serum-S1P levels were not different before CPB starts, the most drastic drop was observed after administration of heparin. ns, not significant, ***p < 0.001. CBP, Cardio pulmonary bypass; ICU, intensive care unit; POD, postoperative day; ns, not significant.

To further define the time window in which S1P concentrations drop during treatment, S1P levels were measured every 30 min in 6 patients during CPB. In all cases, a significant drop of S1P levels was observed immediately before the start of CPB, which is in coincidence with the application of heparin (**Figure 5**).

## Discussion

This report analyses S1P levels in cardiac surgery induced inflammation. Our main findings are that S1P levels are disrupted by heart surgery, changes of S1P levels inversely correlate with acute phase markers for inflammation, and the influence of CPB on S1P levels seems negligible compared to off-pump procedures. Cardiac surgery is an extremely strong inflammatory stimulus induced by tissue trauma, contact activation of the blood with non-endothelial surfaces, coagulation activation, endotoxemia and ischaemia-reperfusion injury (5, 27). This provokes an immediate acute-phase response, which is characterized by alterations of cytokines levels and acute-phase proteins (28, 29). Clinically, the resulting systemic inflammatory response is associated with severe intra- and postoperative complications as myocardial, respiratory and acute kidney injury (AKI), neurological impairment, bleeding and in its most severe form multiorgan failure (27). A recently published systematic review, including data of more than 14,000 patients, analysed which markers were useful in determining the magnitude of surgery induced injury (30). For example, the authors found that peak CRP was 36-times higher in patients after cardiac surgery compared to pre-surgery and that high concentrations of IL-6 and CRP were closely associated with the degree of surgical injury (30). PCT is another well-described marker, but rather suitable for postoperative infectious complications after cardiac surgery (31). Elevated levels of IL-6, CRP, leucocytes and PCT indicate a substantial inflammatory reaction during and after surgery in our cohort. Inflammation in cardiac surgery is often attributed to CPB and there is a still on-going scientific debate on whether operative strategies avoiding CPB may be superior. In our study there was no significant difference in the measured parameters of inflammatory response in both patient groups with and without CPB. Additionally, we did not observe a relevant difference in S1P levels between the two groups and S1P levels are altered in all patients regardless of the use of CPB. In contrast to the clinically established inflammatory markers IL-6, CRP or leukocyte counts, S1P may better indicate a disturbed immune response together with endothelial cell injury. Our data suggests that there is a reliable and prompt S1P drop after the surgical stimulus. The time from the surgical stimulus until the significant S1P drop is very short. Therefore, we believe that, due to the important role of circulating S1P in stabilizing endothelial integrity and immune response, S1P may function as a potential marker of endothelial injury during and after cardiac surgery, which might be predictive for recovery.

A hallmark of systemic inflammation is the damage to the endothelial cell layer and recovery of endothelial cell function determines the outcome. This is most relevant in systemic reactions induced by bacterial pathogens such as in sepsis (32). Cardiac surgery induces a sepsis-like syndrome associated with a high degree of endothelial cell dysfunction. Recent studies report drastically increased postoperative levels of endothelial markers in blood such as vascular endothelial cadherin and endocan, which correlate with the severity of endothelial cell injury after cardiac surgery (33, 34). Von-Willebrand factor regulates adhesion of immune cells to injured endothelium and has been previously studied in cardiac patients (35). In contrast to components released by injured endothelial cells, S1P is a signalling molecule regulating endothelial cell structure *via* specific G-protein coupled receptors (36). The endothelium is an important source of circulatory S1P and maintains cell function in an auto-protective manner (37). Cardiac surgery may interrupt this balance and injured endothelial cells may produce less S1P, which further promotes endothelial dysfunction and barrier breakdown. An analogue mechanism has been discussed by other researchers for chronic endothelial dysfunction in atherosclerosis (38). Furthermore, circulating S1P levels are not only determined by endothelial cells. Hematopoietic cells, erythrocytes and platelets are a rich source for S1P as well and all contribute to plasma-S1P levels (15). Cardiac surgery induces haemolysis; bleeding complications are common and high platelet turnover and microthrombi are frequent. This could be reason for the observed acute changes in S1P concentration during surgery when hematopoietic S1P sources are compromised. Circulating S1P further depends on its carrier proteins HDL and albumin, which contribute to S1P related effects (39). During recovery after surgical trauma healing mechanisms, such as increased production of anti-inflammatory proteins as HDL or albumin, may be involved in restoring S1P homeostasis and explain our observation.

Another function of S1P is its protective role in hyperinflammatory states. Experimental studies have been performed in various models. For instance, in mice infected with H1N1 influenza, administration of S1P receptor 1 agonists attenuate the cytokine release by pulmonary endothelial cells (17). Another well-studied role for S1P is the regulation of lymphocyte egress from lymphatic organs into the blood (14). It is possible, due to the lack of the immunoregulative function of S1P in the perioperative phase following cardiac surgery, that patients are vulnerable for infectious postoperative complications. Even though it is a comparably small cohort with a short observational period of only 4 days, we have found that patients with S1P levels not recovering to preoperative levels stayed longer on the ICU and had a higher SOFA score on POD1.

S1P levels dropped in every individual. The analysis of S1P kinetics revealed the most relevant decrease before the onset of CPB and before equivalent off-pump-procedures, respectively. The decrease in S1P coincided with invasive surgical measures as sternotomy, luxation of the heart and cannulation for CPB and the administration of unfractionated heparin in a high dose to prepare for upcoming CPB or off-pump procedure. According to the standard institutional protocol, on- and off-pump patients receive an initial i.v. bolus of 300-400 IE/kg heparin and one possibility is that S1P levels are influenced by heparin. Yatomi et al. measured 60% lower S1P levels in non-coagulated blood (191 ± 79 vs. 484 ± 82 pmol/mL) (40). Thus, it is possible that heparin is causing the drop of S1P. The release of S1P by thrombin-activated platelets into the circulation has previously been described (41, 42). In trauma, tissue factor (TF) is exposed and initiates the coagulation cascade. Binding of activated factor (F) VII to TF allows the binding of FX and its conversion to FXa, which than stimulates thrombin generation (43). Consequently, targeting FXa, *via* heparin, may attenuate platelet activation and subsequently cause S1P levels to drop. The role of platelet aggregation inhibitors in S1P release by platelets has recently been investigated in pre-clinical studies. The release of S1P induced by activation of thrombin receptor (PAR-1) is inhibited by aspirin *in-vitro* and *ex-vivo* (44). Moreover, FXa induces the expression of S1P producing kinases and subsequently increases S1P formation (45). Taken together, FXa inhibitors, such as heparin, may decrease circulating S1P levels, which has been lately included in a US patent description (US20170296549A1). Mechanistically, this could be an effect of inhibited thrombin activation of platelets, which is a main source for circulating S1P. Reduced S1P levels in open heart surgery might be affected by injured endothelial cells, reduced hematopoietic sources and carriers or by iatrogenic inhibition of the coagulation system. Interestingly, this is the first report showing that high dose anticoagulation with heparin may influence S1P levels in patients undergoing cardiac surgery. We cannot exclude that this hypothetical pleiotropic effect of heparin might have a larger effect on vascular- and immunomodulatory S1P than we expected. A S1P/heparin interaction on the immune response has been described and therefore cannot be excluded also under high-dose heparin treatment during cardiac surgery (46).

Limitations of this study are that it was carried out at a single center and involved a relatively small number of patients. The results are strictly observational and therefore, the identity of underlying mechanisms remains speculative. Nevertheless, due to the reliable drop of S1P in all patients, we believe that our observations warrant follow-up studies. For instance, confirmatory studies are needed to evaluate a possible relationship of high dose heparin with the drop of S1P levels. The observed drop of S1P is robust, however, we do not know if the observed lower S1P levels will have a functional significance on S1P signalling. Follow-up interventional studies should address the potential of S1P or S1P mimics to benefit patients undergoing cardiac surgery.

## Conclusion

In conclusion, in this prospective observational study we report a severe drop in circulatory S1P in patients undergoing cardiac surgery independent of the use of CBP. S1P concentrations might be negatively affected by endothelial injury, loss of S1P sources or other intraoperative events such as anticoagulation by heparin. It is an intriguing possibility to utilize measurements of circulatory S1P to predict the severity of surgery-induced inflammation, ICU length of stay and general clinical outcome. Moreover, our observations encourage future interventional studies to investigate the therapeutical potential of S1P or S1P mimics in cardiac surgery.

## Data Availability Statement

The raw data supporting the conclusions of this article will be made available by the authors, without undue reservation.

## Ethics Statement

The studies involving human participants were reviewed and approved by Ethics committee - Aerztekammer Hamburg. The patients/participants provided their written informed consent to participate in this study.

## Author Contributions

Study conception and design: GG and MW. Acquisition of data: GG, EM, HS, AP, AN, ES, and MW. Analysis and interpretation of data: GG, EM, KA, ES, GD, BT, and MW. Drafting manuscript: GG, ES, KA, GD, and MW. Critical revision: GG, AN, SK, HR, CZ, ES, GD, BT, and MW. All authors contributed to the article and approved the submitted version.

## Funding

The study was financed by internal institutional funding.

## Conflict of Interest

SK declares the following competing interests: He received research support by Ambu, E.T.View Ltd, Fisher & Paykel, Pfizer and Xenios; lecture honorarium from Arjo-Huntleigh, Astellas, Astra, Basilea, Bard, Baxter, Biotest, CSL Behring, Cytosorbents, Fresenius, Gilead, MSD, Orion, Pfizer, Philips, Sedana, Sorin, Xenios and Zoll. SK received consultant honorarium from AMOMED, Astellas, Baxter, Bayer, Fresenius, Gilead, MSD, Pfizer and Xenios. MW declares the following competing interests of funding from Sartorius lung research.

The remaining authors declare that the research was conducted in the absence of any commercial or financial relationships that could be construed as a potential conflict of interest.

## Publisher’s Note

All claims expressed in this article are solely those of the authors and do not necessarily represent those of their affiliated organizations, or those of the publisher, the editors and the reviewers. Any product that may be evaluated in this article, or claim that may be made by its manufacturer, is not guaranteed or endorsed by the publisher.
